# Supplementary material for: In vivo dissection of a clustered-CTCF domain boundary reveals developmental principles of regulatory insulation
Source: Nat Genet. 2022 Jul 11;54(7):1026–36. doi: 10.1038/s41588-022-01117-9 (PMC9279147; doi:10.1038/s41588-022-01117-9)
Supplement: Supplementary file 1 — Supplementary Figs. 1–5. [file 41588_2022_1117_MOESM1_ESM.pdf]

nature portfolio

https://doi.org/10.1038/s41588-022-01117-9

Supplementary information

In vivo dissection of a clustered-CTCF domain boundary reveals developmental principles of regulatory insulation

In the format provided by the authors and unedited

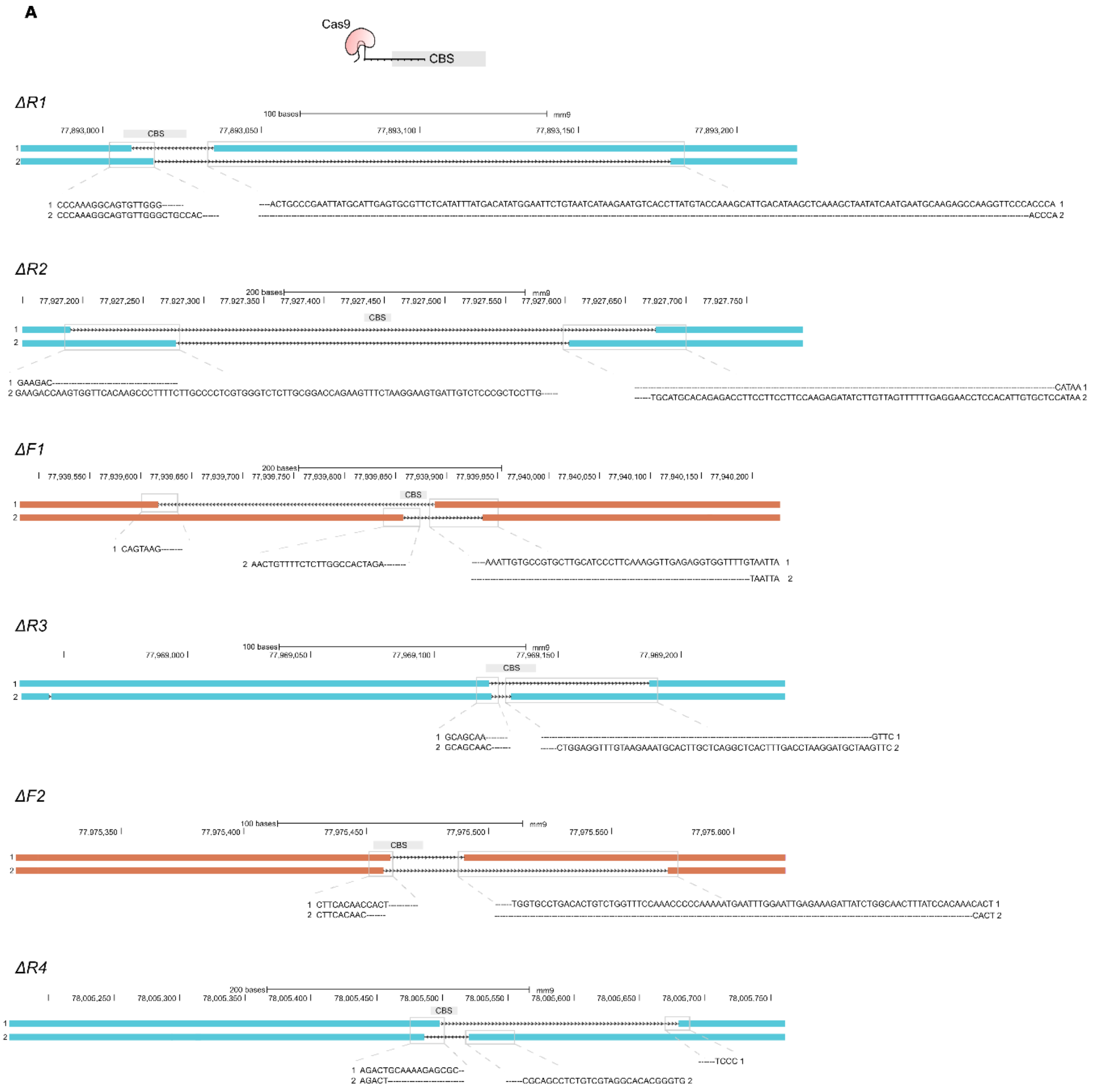

Supp. Fig. 1 *CBS individual deletions generated by CRISPR/Cas9.*

A. Sanger sequencing reads schematics showing the deletion profiles for the six individual CBS mutants for the two alleles. CBS positions are specified. Deleted allelic regions are represented as black dashed lines. Intact allelic regions are indicated with light blue (reverse CBS) and orange (forward CBS) bars. Close-ups show the exact breakpoints sequences.

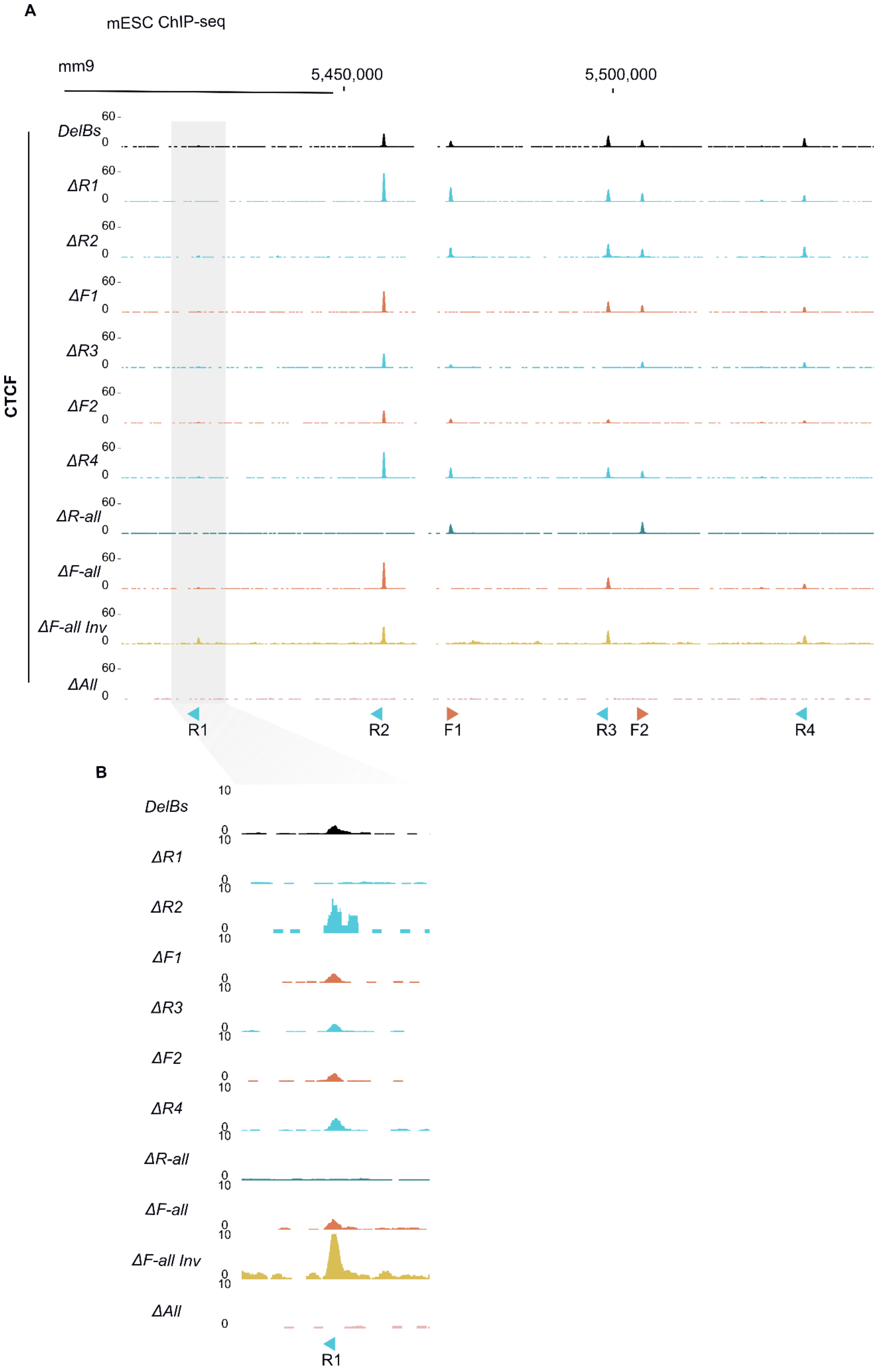

Supp. Fig. 2. *Targeted CBS deletions completely abrogate CTCF binding in the intended loci.*

A. CTCF ChIP-seq experiments performed in the different mESC mutant clones used for tetraploid complementation. The ChIP-seq tracks show a complete absence of CTCF binding on the mutated CBS. The locations of the WT CBS are depicted with red and blue arrowheads (forward (F) and reverse (R) oriented respectively). Note that R1 and R2 in mESC display different binding enrichment as in limb data (**Fig. 1B**), denoting tissue-specific variation. **B.** Zoom in of the R1 genomic region. Note the absence of CTCF binding in mutants with deleted R1 CBS

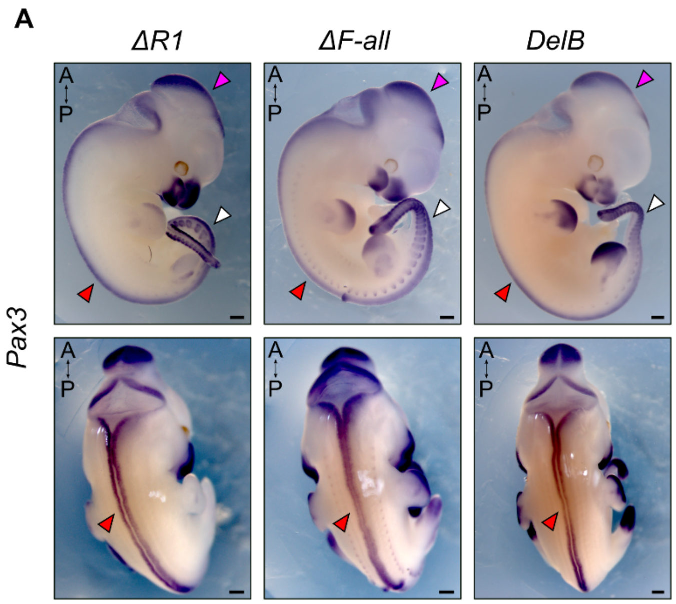

Supp. Fig. 3 *Invariant Pax3 expression pattern in different tissues.*

A. WISH shows *Pax3* expression in E11.5 mutants. Arrowheads indicate midbrain (purple), tail somites (white) and spinal cord (red). Note how the pattern of expression of *Pax3* is preserved in all tissues with the exception of the limb bud in the different CBS mutants. A, anterior; P, posterior. Scale bar: 500µm.

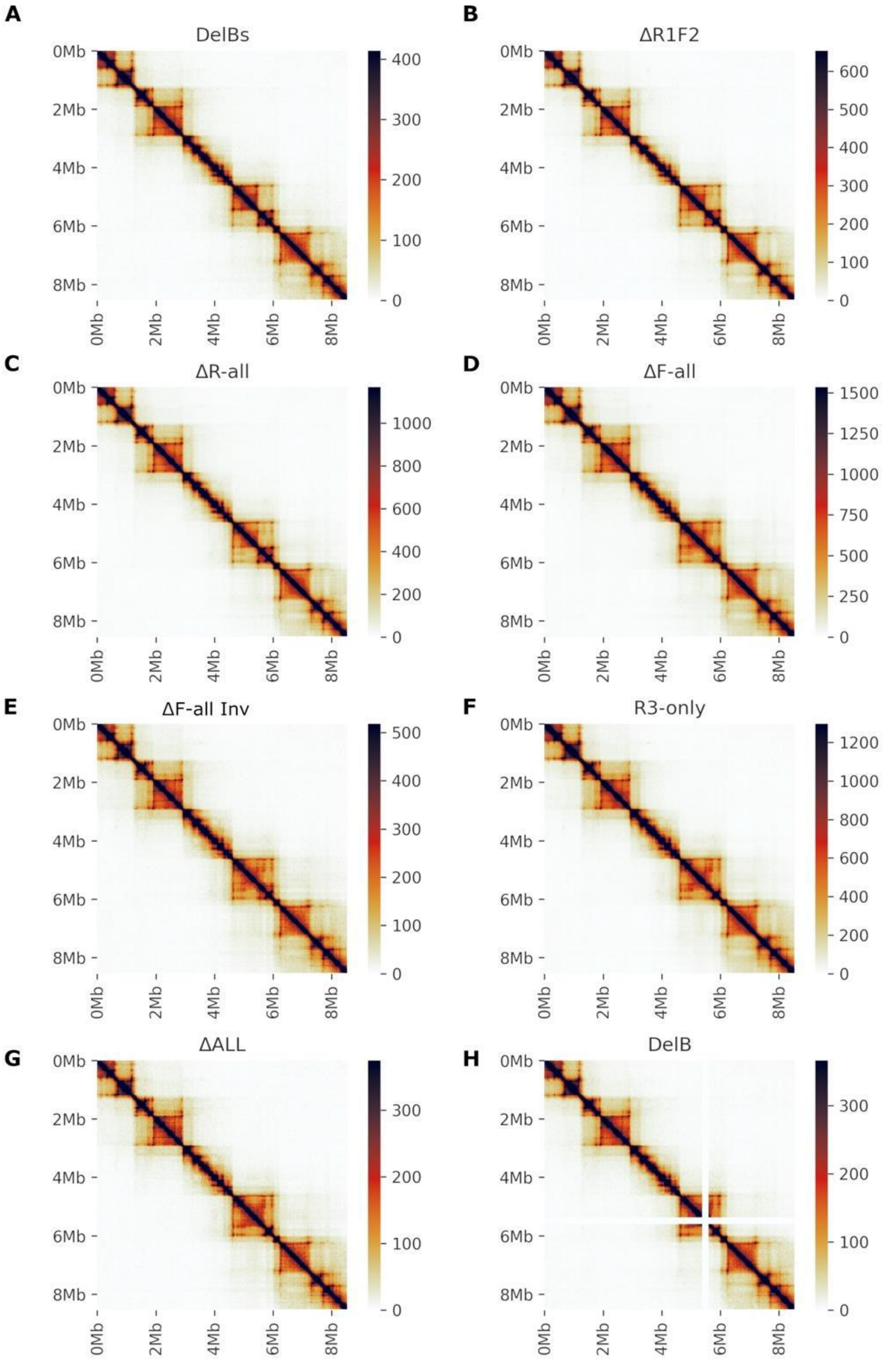

Supp. Fig. 4 *General view of the interaction matrices of embryonic distal limb cHi-C experiments*

A-H. Interaction matrices from embryonic limb cHi-C aligned to the custom genome representing the captured region (~8,3 Mb corresponding to the mm9 coordinates 71-81Mb excluding the *Epha4* intra-TAD deletion carried by the baseline *DelBs* mutant). The only exception is E (*ΔF-all Inv* mutant), where a modified version of the custom genome is used to account for the inversion of the boundary. Changes in the pattern of interactions of the different mutants are restricted to the progressive fusion of the *Epha4* and the *Pax3* TADs (black arrowheads).

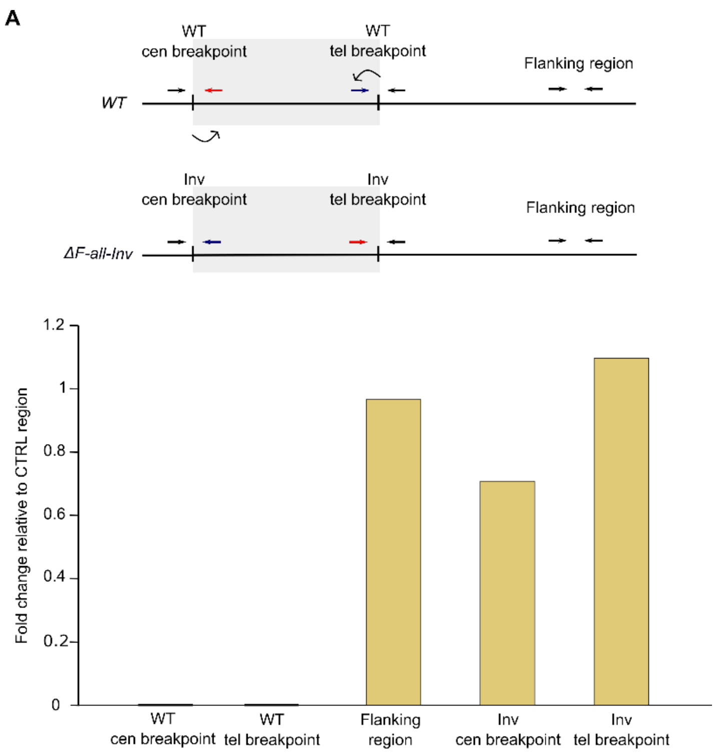

Supp. Fig. 5 *ΔF-all-Inv genotyping.*

A. Schematics showing primer pairs positions (upper panel) and corresponding qPCR quantifying copy number of the inverted region in mutant mouse embryonic stem cells (lower panel). Bars represent the mean of three technical replicates. Values are normalized on a CTRL region on Chr13. Note how the *ΔF-all-Inv* mutant does not show any copy of the WT breakpoints. Cen, centromeric. Tel, telomeric
