# Supplementary material for: In vivo dissection of a clustered-CTCF domain boundary reveals developmental principles of regulatory insulation
Source: Nat Genet. 2022 Jul 11;54(7):1026–36. doi: 10.1038/s41588-022-01117-9 (PMC9279147; doi:10.1038/s41588-022-01117-9)
Supplement: Supplementary file 2 — Reporting Summary [file 41588_2022_1117_MOESM2_ESM.pdf]

nature research

Corresponding author(s): Darío Lupiáñez

Last updated by author(s): 2022/04/18

Reporting Summary

Nature Research wishes to improve the reproducibility of the work that we publish. This form provides structure for consistency and transparency in reporting. For further information on Nature Research policies, see our Editorial Policies and the Editorial Policy Checklist.

Statistics

For all statistical analyses, confirm that the following items are present in the figure legend, table legend, main text, or Methods section.

n/a	Confirmed	
☐	☒	The exact sample size (*n*) for each experimental group/condition, given as a discrete number and unit of measurement
☒	☐	A statement on whether measurements were taken from distinct samples or whether the same sample was measured repeatedly
☐	☒	The statistical test(s) used AND whether they are one- or two-sided<br>*Only common tests should be described solely by name; describe more complex techniques in the Methods section.*
☒	☐	A description of all covariates tested
☐	☒	A description of any assumptions or corrections, such as tests of normality and adjustment for multiple comparisons
☐	☒	A full description of the statistical parameters including central tendency (e.g. means) or other basic estimates (e.g. regression coefficient) AND variation (e.g. standard deviation) or associated estimates of uncertainty (e.g. confidence intervals)
☐	☒	For null hypothesis testing, the test statistic (e.g. *F*, *t*, *r*) with confidence intervals, effect sizes, degrees of freedom and *P* value noted<br>*Give P values as exact values whenever suitable.*
☒	☐	For Bayesian analysis, information on the choice of priors and Markov chain Monte Carlo settings
☒	☐	For hierarchical and complex designs, identification of the appropriate level for tests and full reporting of outcomes
☐	☒	Estimates of effect sizes (e.g. Cohen's *d*, Pearson's *r*), indicating how they were calculated

Our web collection on statistics for biologists contains articles on many of the points above.

Software and code

Policy information about availability of computer code

Data collection	No software was used for data collection.
Data analysis	Custom code is available in a gitlab repository (https://gitlab.com/rdacemel/anania2021).<br><br>The following publicly available programs were used:<br>bwa (v0.7.17-r1188)<br>pairtools (v0.3.0)<br>juicertools (v1.22.01)<br>fanc (v0.9.17).<br>bowtie (v1.2.3)<br>samtools (v1.9)<br>bedtools (v2.29.2)<br>bedGraphToBigWig (kentUtils v4)<br>FIMO (MEME, v5.1.1)<br>deeptools (v3.5.1)<br><br>The following python==3.7.10 package versions were used:<br>pandas==1.2.3<br>numpy==1.20.1<br>pybedtools==0.8.2<br>seaborn==0.11.1<br>matplotlib==3.3.3<br>scikit-learn==0.24.1

scipy==1.6.1
statsmodels==0.12.2
scikit-posthocs==0.6.6

The following R==4.1.0 packages versions were used:
dplyr==1.0.7
data.table==1.14.0
ggplot2==3.3.2

For manuscripts utilizing custom algorithms or software that are central to the research but not yet described in published literature, software must be made available to editors and reviewers. We strongly encourage code deposition in a community repository (e.g. GitHub). See the Nature Research guidelines for submitting code & software for further information.

Data

Policy information about availability of data

All manuscripts must include a data availability statement. This statement should provide the following information, where applicable:

- Accession codes, unique identifiers, or web links for publicly available datasets
- A list of figures that have associated raw data
- A description of any restrictions on data availability

Sequencing data from cHi-C, ChIPmentation and ChIP-seq experiments is available in GEO (GSE169561).

Field-specific reporting

Please select the one below that is the best fit for your research. If you are not sure, read the appropriate sections before making your selection.

☒ Life sciences ☐ Behavioural & social sciences ☐ Ecological, evolutionary & environmental sciences

For a reference copy of the document with all sections, see nature.com/documents/nr-reporting-summary-flat.pdf

Life sciences study design

All studies must disclose on these points even when the disclosure is negative.

Sample size	Sample size was at least 3 homozygous animals for qPCR and WISH. Phenotypical analysis on mutant embryos were performed for at least 4 homozygous animals. cHi-C experiments were performed in single-replicates using a pool of distal limbs from different embryos. These are commonly accepted sample sizes for these types of experiments (Franke et al., Nature, 2016; Bianco et al., Nature Genetics 2018; Rouco et al., Nature Communications, 2021). Rad21 ChIPmentation experiments were performed in duplicates as is the standard set by the ENCODE project for this type of experiments. CTCF ChIP-seq experiments were performed in one replicate since they were only used as a control to certify the total absence of CTCF binding upon CTCF binding site deletions in the different mutant cell lines
Data exclusions	Samples were excluded only according to the genotype which was analysed in control experiments.
Replication	At least 3 embryos with homozygous genotype were used to perform WISH experiments and they gave reproducible staining. At least 4 embryos were used to perform skeletal staining and phenotypical analysis giving reproducible results. Statistical analysis was performed on the measurements of the finger lengths which are stated in the figure legend of Main Fig. 6. Individual embryos datapoint for the finger lengths are presented in Main Fig. 6. At least 3 embryos were used to perform qPCR experiments, individual datapoints are presented in the figures. Statistical analysis was performed on the qPCR experiments which is stated in the manuscript and in the figure legends. Reproducibility of single replicate cHi-C experiments was found to be high outside from the two TADs of interest (proposed in Bianco et al., Nature Genetics 2018; Pearson > 0.89, see more details in Methods). Rad21 ChIPmentation experiments were performed in duplicates as is the standard set by the ENCODE project for this type of experiments with the exception of R3-only and R1+F2 genotypes due to difficulties obtaining enough embryonic material from these genetic backgrounds. CTCF ChIP-seq experiments were performed in one replicate since they were only used as a control to certify the total absence of CTCF binding upon CTCF binding site deletions in the different mutant cell lines
Randomization	Randomization is not relevant on this study because all the experiments performed had to take into account the genotype of the samples and there is no treatment involved nor additional covariates expected to introduce biases in development.
Blinding	The experiments were not performed blindly because the embryos generation and analysis required knowledge about their genotype and all comparisons were performed automatically using statistical software that is not influenced by the investigator.

Reporting for specific materials, systems and methods

We require information from authors about some types of materials, experimental systems and methods used in many studies. Here, indicate whether each material, system or method listed is relevant to your study. If you are not sure if a list item applies to your research, read the appropriate section before selecting a response.

Materials & experimental systems

n/a	Involved in the study
☐	☒ Antibodies
☐	☒ Eukaryotic cell lines
☒	☐ Palaeontology and archaeology
☐	☒ Animals and other organisms
☒	☐ Human research participants
☒	☐ Clinical data
☒	☐ Dual use research of concern

Methods

n/a	Involved in the study
☐	☒ ChIP-seq
☒	☐ Flow cytometry
☒	☐ MRI-based neuroimaging

Antibodies

Antibodies used

CTCF-Ab Diagenode Cat. No. C15410210 Lot. No. A2359-00234D (1ug per IP) also stated in Material and Methods
RAD21-Ab ABCAM Cat. No. ab992 Lot. N0. GR3310168 (4ug per IP) also stated in Material and Methods
Anti-Digoxigenin-AP, Fab fragments ROCHE Cat. No. 11093274910 (used 1:5000) also stated in Material and Methods

Validation

CTCF-Ab
Polyclonal ChIP-seq grade. Species reactivity: human and mice. Host: Rabbit. Applications: ChIP/ChIP-seq, ELISA, Western Blotting, Immunofluorescence. Validation by the manufacturer: determination of Ab titer by ELISA; validation in HeLa cells by ChIP-qPCR, ChIP-seq, Western Blot and Immunofluorescence.
RAD21-Ab
Polyclonal. Species reactivity: human and mice. Host: Rabbit. Applications: Immunoprecipitation and Western Blotting. Validation by the manufacturer: validation by Immunoprecipitation in Hep3B human cell lysate; validation by Western Blot in different mouse and human cell lysate.
Anti-Digoxigenin-AP, Fab fragments
Polyclonal anti-digoxigenin antibodies. Host: sheep. Applications: cDNA array, Colony/plaque hybridization, Dot blot, ELISA, Gel shift assay, Immunohistocytochemistry, In situ hybridization, Nonradioactive DNA sequencing blot, Northern blot, RNase protection assay, Southern blot, Western blot, Fluorescent in situ hybridization, Section in situ hybridization and whole mount in situ hybridization, Electrophoretic mobility shift assay. Validation by the manufacturer by Dot blot, ELISA, Immunohistocytochemistry, In situ hybridization, Southern blot and Western blot.

Eukaryotic cell lines

Policy information about cell lines

Cell line source(s)

G4F1 (https://doi.org/10.1073/pnas.0609277104)
G4F1 /DelBs
G4F1 /DelB
G4F1 /ΔR1- ΔR2- ΔR3- ΔR4- ΔF1- ΔF2
G4F1 /ΔR1+F2
G4F1 /ΔR-all
G4F1 /ΔF-all
G4F1/ΔF-all-Inv
G4F1/ΔALL
G4F1/R3-only

Authentication

Cell-lines were not authenticated

Mycoplasma contamination

All cells were tested for mycoplasma contamination using Mycoalert detection kit (Lonza) and Mycoalert assay control set (Lonza)

Commonly misidentified lines
(See ICLAC register)

No commonly misidentified lines cell lines were used.

Animals and other organisms

Policy information about studies involving animals; ARRIVE guidelines recommended for reporting animal research

Laboratory animals

Mus musculus, CD-1, female, various ages for donor and embryos retransfered by tetraploid aggregation and E11.5 and E17.5 embryos isolated to perform experimental analysis.

Wild animals

This study did not involve wild animals.

Field-collected samples

This study did not involve field-collected samples.

Ethics oversight

Mice were handled according to institutional guidelines under an experimentation license (G0111/17) approved by the Landesamt fuer Gesundheit und Soziales (Berlin, Germany)

Note that full information on the approval of the study protocol must also be provided in the manuscript.

ChIP-seq

Data deposition

☒ Confirm that both raw and final processed data have been deposited in a public database such as GEO.

☒ Confirm that you have deposited or provided access to graph files (e.g. BED files) for the called peaks.

Data access links
May remain private before publication.

Sequencing data from cHi-C, ChIPmentation and ChIP-seq experiments is available in GEO (GSE169561).

Files in database submission

ChIP_CTCF_mESC_DelBs.fq.gz ChIP_CTCF_mESC_DelBs.bw
ChIP_CTCF_mESC_F1.fq.gz ChIP_CTCF_mESC_F1.bw
ChIP_CTCF_mESC_F2.fq.gz ChIP_CTCF_mESC_F2.bw
ChIP_CTCF_mESC_F-ALL.fq.gz ChIP_CTCF_mESC_F-ALL.bw
ChIP_CTCF_mESC_F-ALL-INV.fq.gz ChIP_CTCF_mESC_F-ALL-INV.bw
ChIP_CTCF_mESC_R1.fq.gz ChIP_CTCF_mESC_R1.bw
ChIP_CTCF_mESC_R2.fq.gz ChIP_CTCF_mESC_R2.bw
ChIP_CTCF_mESC_R3.fq.gz ChIP_CTCF_mESC_R3.bw
ChIP_CTCF_mESC_R4.fq.gz ChIP_CTCF_mESC_R4.bw
ChIP_CTCF_mESC_R-ALL.fq.gz ChIP_CTCF_mESC_R-ALL.bw
ChIP_CTCF_mESC_ALL.fq.gz ChIP_CTCF_mESC_ALL.bw
ChIP_CTCF_DistalLimb_E11-5_WT_r1.fq.gz ChIP_CTCF_DistalLimb_E11-5_WT_r1.bw
ChIP_Rad21_DistalLimb_E11-5_ALL_r1.fq.gz ChIP_Rad21_DistalLimb_E11-5_ALL_r1.bw
ChIP_Rad21_DistalLimb_E11-5_ALL_r1_input.fq.gz ChIP_Rad21_DistalLimb_E11-5_ALL_r1_input.bw
ChIP_Rad21_DistalLimb_E11-5_ALL_r2.fq.gz ChIP_Rad21_DistalLimb_E11-5_ALL_r2.bw
ChIP_Rad21_DistalLimb_E11-5_ALL_r2_input.fq.gz ChIP_Rad21_DistalLimb_E11-5_ALL_r2_input.bw
ChIP_Rad21_DistalLimb_E11-5_DelBs_r1.fq.gz ChIP_Rad21_DistalLimb_E11-5_DelBs_r1.bw
ChIP_Rad21_DistalLimb_E11-5_DelBs_r2.fq.gz ChIP_Rad21_DistalLimb_E11-5_DelBs_r2.bw
ChIP_Rad21_DistalLimb_E11-5_DelBs_r2_input.fq.gz ChIP_Rad21_DistalLimb_E11-5_DelBs_r2_input.bw
ChIP_Rad21_DistalLimb_E11-5_FALLinv_r1.fq.gz ChIP_Rad21_DistalLimb_E11-5_FALLinv_r1.bw
ChIP_Rad21_DistalLimb_E11-5_FALLinv_r1_input.fq.gz ChIP_Rad21_DistalLimb_E11-5_FALLinv_r1_input.bw
ChIP_Rad21_DistalLimb_E11-5_FALLinv_r2.fq.gz ChIP_Rad21_DistalLimb_E11-5_FALLinv_r2.bw
ChIP_Rad21_DistalLimb_E11-5_FALLinv_r2_input.fq.gz ChIP_Rad21_DistalLimb_E11-5_FALLinv_r2_input.bw
ChIP_Rad21_DistalLimb_E11-5_FALL_r1.fq.gz ChIP_Rad21_DistalLimb_E11-5_FALL_r1.bw
ChIP_Rad21_DistalLimb_E11-5_FALL_r1_input.fq.gz ChIP_Rad21_DistalLimb_E11-5_FALL_r1_input.bw
ChIP_Rad21_DistalLimb_E11-5_FALL_r2.fq.gz ChIP_Rad21_DistalLimb_E11-5_FALL_r2.bw
ChIP_Rad21_DistalLimb_E11-5_FALL_r2_input.fq.gz ChIP_Rad21_DistalLimb_E11-5_FALL_r2_input.bw
ChIP_Rad21_DistalLimb_E11-5_R1F2_r1.fq.gz ChIP_Rad21_DistalLimb_E11-5_R1F2_r1.bw
ChIP_Rad21_DistalLimb_E11-5_R1F2_r1_input.fq.gz ChIP_Rad21_DistalLimb_E11-5_R1F2_r1_input.bw
ChIP_Rad21_DistalLimb_E11-5_R3-only_r1.fq.gz ChIP_Rad21_DistalLimb_E11-5_R3-only_r1.bw
ChIP_Rad21_DistalLimb_E11-5_R3-only_r1_input.fq.gz ChIP_Rad21_DistalLimb_E11-5_R3-only_r1_input.bw
ChIP_Rad21_DistalLimb_E11-5_RALL_r1.fq.gz ChIP_Rad21_DistalLimb_E11-5_RALL_r1.bw
ChIP_Rad21_DistalLimb_E11-5_RALL_r1_input.fq.gz ChIP_Rad21_DistalLimb_E11-5_RALL_r1_input.bw
ChIP_Rad21_DistalLimb_E11-5_RALL_r2.fq.gz ChIP_Rad21_DistalLimb_E11-5_RALL_r2.bw
ChIP_Rad21_DistalLimb_E11-5_RALL_r2_input.fq.gz ChIP_Rad21_DistalLimb_E11-5_RALL_r2_input.bw
ChIP_Rad21_DistalLimb_E11-5_WT_r1.fq.gz ChIP_Rad21_DistalLimb_E11-5_WT_r1.bw

Genome browser session
(e.g. UCSC)

https://genome.mdc-berlin.de/cgi-bin/hgTracks?
hgS_doOtherUser=submit&hgS_otherUserName=rdacemel&hgS_otherUserSessionName=DelBs_public

Methodology

Replicates

One replicate was used for CTCF ChIP-seq experiments were used as a control to certify the total absence of CTCF binding upon CTCF binding site deletions in the different mutant cell lines. Rad21 ChIPmentation experiments were performed in duplicates as is the standard set by the ENCODE project for this type of experiments with the exception of R3-only and R1+F2 genotypes due to difficulties obtaining enough embryonic material from these genetic backgrounds.

Sequencing depth

ChIP_CTCF_mESC_DelBs // TOTAL: 53825880 // ALIGNED: 41999899 // LENGTH: 75bp // SINGLE-END
ChIP_CTCF_mESC_F1 // TOTAL: 53085169 // ALIGNED: 38545675 // LENGTH: 75bp // SINGLE-END
ChIP_CTCF_mESC_F2 // TOTAL: 51452800 // ALIGNED: 36878701 // LENGTH: 75bp // SINGLE-END
ChIP_CTCF_mESC_F-ALL // TOTAL: 44551297 // ALIGNED: 30035416 // LENGTH: 75bp // SINGLE-END
ChIP_CTCF_mESC_F-ALL-INV // TOTAL: 67485877 // ALIGNED: 46882387 // LENGTH: 75bp // SINGLE-END
ChIP_CTCF_mESC_R1 // TOTAL: 48777397 // ALIGNED: 33726017 // LENGTH: 75bp // SINGLE-END
ChIP_CTCF_mESC_R2 // TOTAL: 33328381 // ALIGNED: 19947596 // LENGTH: 75bp // SINGLE-END
ChIP_CTCF_mESC_R3 // TOTAL: 58086689 // ALIGNED: 43324716 // LENGTH: 75bp // SINGLE-END
ChIP_CTCF_mESC_R4 // TOTAL: 44628642 // ALIGNED: 30282667 // LENGTH: 75bp // SINGLE-END
ChIP_CTCF_mESC_R-ALL // TOTAL: 85948593 // ALIGNED: 66275753 // LENGTH: 75bp // SINGLE-END
ChIP_CTCF_mESC_ALL // TOTAL: 37638816 // ALIGNED: 20326201 // LENGTH: 75bp // SINGLE-END
ChIP_CTCF_DistalLimb_E11-5_WT_r1 // TOTAL:58028751 // ALIGNED:19189393 // LENGTH:75bp// SINGLE-END
ChIP_Rad21_DistalLimb_E11-5_ALL_r1 // TOTAL:58009791 // ALIGNED:45921400 // LENGTH:75bp// SINGLE-END
ChIP_Rad21_DistalLimb_E11-5_ALL_r1_input // TOTAL:62815605 // ALIGNED:40443783 // LENGTH:100bp// SINGLE-END

ChIP_Rad21_DistalLimb_E11-5_ALL_r2 // TOTAL:40093948 // ALIGNED:30936732 // LENGTH:100bp // SINGLE-END
ChIP_Rad21_DistalLimb_E11-5_ALL_r2_input // TOTAL:41267431 // ALIGNED:41267431 // LENGTH:100bp // SINGLE-END
ChIP_Rad21_DistalLimb_E11-5_DelBs_r1 // TOTAL:50738061 // ALIGNED:37249790 // LENGTH:100bp // SINGLE-END
ChIP_Rad21_DistalLimb_E11-5_DelBs_r2 // TOTAL:55999774 // ALIGNED:41326409 // LENGTH:100bp // SINGLE-END
ChIP_Rad21_DistalLimb_E11-5_DelBs_r2_input // TOTAL:43255640 // ALIGNED:14296889 // LENGTH:100bp // SINGLE-END
ChIP_Rad21_DistalLimb_E11-5_FALLinv_r1 // TOTAL:56707612 // ALIGNED:45972724 // LENGTH:75bp // SINGLE-END
ChIP_Rad21_DistalLimb_E11-5_FALLinv_r1_input // TOTAL:42628140 // ALIGNED:29835034 // LENGTH:100bp // SINGLE-END
ChIP_Rad21_DistalLimb_E11-5_FALLinv_r2 // TOTAL:39116675 // ALIGNED:24580837 // LENGTH:100bp // SINGLE-END
ChIP_Rad21_DistalLimb_E11-5_FALLinv_r2_input // TOTAL:39519615 // ALIGNED:19168144 // LENGTH:100bp // SINGLE-END
ChIP_Rad21_DistalLimb_E11-5_FALL_r1 // TOTAL:59839080 // ALIGNED:45181541 // LENGTH:75bp // SINGLE-END
ChIP_Rad21_DistalLimb_E11-5_FALL_r1_input // TOTAL:65997517 // ALIGNED:45522134 // LENGTH:100bp // SINGLE-END
ChIP_Rad21_DistalLimb_E11-5_FALL_r2 // TOTAL:41343217 // ALIGNED:31073011 // LENGTH:100bp // SINGLE-END
ChIP_Rad21_DistalLimb_E11-5_FALL_r2_input // TOTAL:43250838 // ALIGNED:13358619 // LENGTH:100bp // SINGLE-END
ChIP_Rad21_DistalLimb_E11-5_R1F2_r1 // TOTAL:43417607 // ALIGNED:30083365 // LENGTH:100bp // SINGLE-END
ChIP_Rad21_DistalLimb_E11-5_R1F2_r1_input // TOTAL:38118414 // ALIGNED:18379675 // LENGTH:100bp // SINGLE-END
ChIP_Rad21_DistalLimb_E11-5_R3-only_r1 // TOTAL:51110374 // ALIGNED:37618702 // LENGTH:100bp // SINGLE-END
ChIP_Rad21_DistalLimb_E11-5_R3-only_r1_input // TOTAL:46269910 // ALIGNED:14783961 // LENGTH:100bp // SINGLE-END
ChIP_Rad21_DistalLimb_E11-5_RALL_r1 // TOTAL:50527290 // ALIGNED:38180577 // LENGTH:75bp // SINGLE-END
ChIP_Rad21_DistalLimb_E11-5_RALL_r1_input // TOTAL:64021077 // ALIGNED:40262844 // LENGTH:100bp // SINGLE-END
ChIP_Rad21_DistalLimb_E11-5_RALL_r2 // TOTAL:43654593 // ALIGNED:34243430 // LENGTH:100bp // SINGLE-END
ChIP_Rad21_DistalLimb_E11-5_RALL_r2_input // TOTAL:44550398 // ALIGNED:23027998 // LENGTH:100bp // SINGLE-END
ChIP_Rad21_DistalLimb_E11-5_WT_r1 // TOTAL:44702940 // ALIGNED:16362929 // LENGTH:100bp // SINGLE-END

Antibodies

CTCF Ab Diagenode:C15410210 Lot. No. A2359-00234D also stated in Material and Methods
RAD21 Ab (ABCAM) ab992 lot. num. GR3310168 also stated in Material and Methods

Peak calling parameters

No peak calling was performed since the objective of the ChIP-seq experiments was to certify the total absence of CTCF binding upon CTCF binding site deletions in the different mutant cell lines and the cohesin (Rad21) dynamics over a single locus, the EP-boundary.

Data quality

We manually assessed the absence of reads at the deleted CTCF binding sites, and the presence of the expected CTCF enrichment at the remaining and already described CTCF binding sites (see Extended Data Fig. 4). For Rad21 ChIPmentation we assessed the cohesin loading in the EP-boundary and its reproducibility between replicates (see UCSC session).

Software

bowtie v1.2.3
samtools v1.9
bedtools v2.29.2
bedGraphToBigWig (kentUtils) v4
deeptools (v3.5.1)
